# Supplementary figures and images for: Up-regulated IL-17 and Tnf signaling in bone marrow cells of young male osteogenesis imperfecta mice
Source: PeerJ. 2022 Aug 23;10:e13963. doi: 10.7717/peerj.13963 (PMC9415356; doi:10.7717/peerj.13963)

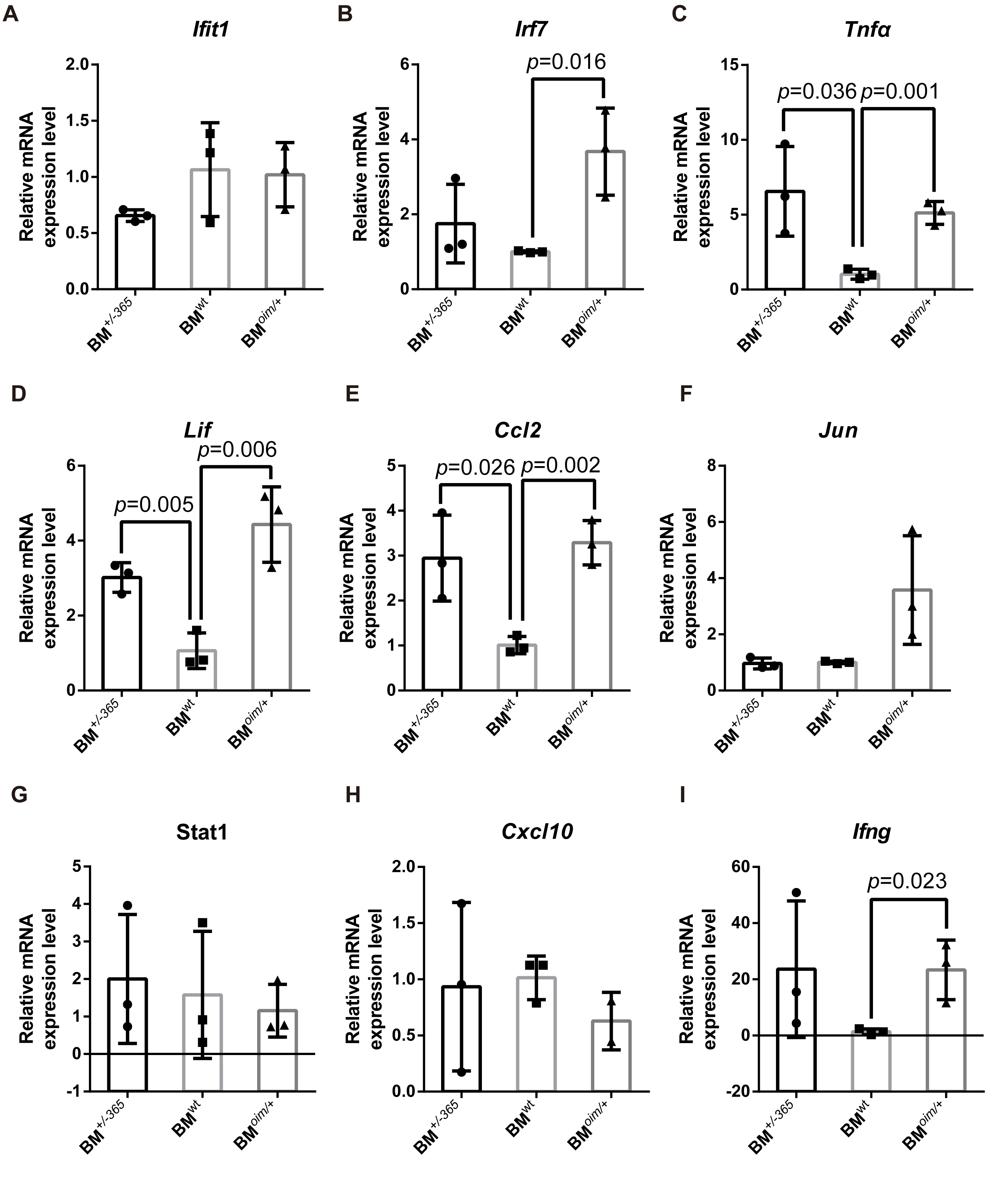

Supplement: Supplemental Information 3 — (A–I) Real-time PCR tested the expression levels of some DEGs. The result indicated that Tnfα (C), Lif (D), and Ccl2 (E) were upregulated in 12-weeks of mutant BM cells (n = 3 for each group, P < 0.05). No obvious expression difference of Ifit1 (A), Jun (F), Stat1 (G) and Cxcl10 (H) in defect OI BM and normal OI BM (n = 3 for each group). Irf7 (B) and Ifng (I) were increased alone in BMoim/+ in 12-weeks (n = 3 for each group, P < 0.05). Data in the quantitative plots are presented as mean ± SD using upaired t-test. [file peerj-10-13963-s003.png]
